# The Effect of Shear Deformation on Permeability of 2.5D Woven Preform

**DOI:** 10.3390/ma12213594

**Published:** 2019-10-31

**Authors:** Zhiming Chen, Shidong Pan, Zhengong Zhou, Tao Lei, Baofeng Dong, Peifei Xu

**Affiliations:** 1 National Key Laboratory of Science and Technology on Advanced Composite in Special Environments, Harbin Institute of Technology, Harbin 150080, China; czm_hit@163.com (Z.C.); zhouzhg@hit.edu.cn (Z.Z.); 1076933369@qq.com (T.L.); 1786122392@qq.com (B.D.); xupeifei@foxmail.com (P.X.); 2 Center for Composite Materials and Structures, Harbin Institute of Technology, Harbin 150001, China

**Keywords:** 2.5D woven preform, permeability, shear deformation

## Abstract

The accurate prediction of the permeability is the key to optimizing the molding process of fiber reinforced composites, thus to improve the composite quality, and reduce the material and labor costs in the manufacturing process. In this paper, the permeability of 2.5D woven preform with shear deformation was studied by experiments and numerical simulations. The permeabilities of the samples under various shear angles were measured by the radial flow method. An RVE (representative volume element) model based on the fabric microstructure and shear deformation is developed to predict the permeability of preform and the simulation results are compared with experiments value to verify the effectiveness of this model. Using this model, the effect of the fiber volume fraction on the permeability of the 2.5D woven preform was determined. Based on the structural characteristics, experimental and simulation results of the 2.5D woven preform, an empirical equation for predicting its permeability under shear deformation was formulated. The prediction accuracy of the equation was evaluated, and the equation was used to determine the change of permeability with shear deformation for the 2.5D woven preform.

## 1. Introduction

Resin transfer molding (RTM) has become an increasingly important technique for the fabrication of the composite materials in the automotive, aviation, and marine industries. The process consists of the following steps: laying of the fiber reinforcement into a mold; injecting the resin; then, curing and lastly, demolding. Through accurate simulation of the process, RTM could be optimized to improve the quality of formed products, to reduce material and labor costs in the manufacturing process. The permeability of dry-fiber reinforcement is found to be one of the key factors affecting the simulation accuracy [1]. Among the numerous fiber reinforcement composites, 2.5D woven preform is a new type of three-dimensional woven reinforcement with good interlaminar properties and impact resistance. It can be woven by machine without requiring manual fiber placement, thereby significantly increasing the production efficiency and lowering the production costs [2,3,4,5,6].

However, due to the complex and diversity of the shapes of the formed structures, the fiber reinforcement is inevitably subjected to local shear stresses, especially when the surface of the target preform is spherical or curved, since the shear deformation would cause the local rearrangement of the fibers and redistribution of fabrics [7,8]. To optimize the formation process, improve the forming quality of the structures, and reduce the material and labor costs in the manufacturing process, it is important to study the effect of shear deformation on the permeability of 2.5D woven preforms.

Scholars have been working for many years to accurately determine the permeability of fiber reinforcements. For example, the Gebart [9] and Kozeny-Carman [10] models were based on the idea that the permeability of the unidirectional fiber reinforcements depends only on the volume fraction of the fibers, and they were used to theoretically predict the permeabilities of unidirectional fiber reinforcements. Endruweit [11] studied the effect of the yarn cross-sectional shape on the resin flow between inter-yarn gaps by combining theoretical analysis with numerical simulations. Endruweit [12] and Swiry [13] studied the permeability of single/multi-layer plain woven fabrics and investigated the influence of the number of layers on the permeability. Chen [14] used a dual-scale model to predict the fabric permeability, and the results showed that the permeabilities of fabrics with the same fiber volume fractions were not necessarily the same. Investigators [15,16,17,18] have established theoretical models to predict the permeabilities of three-dimensional orthogonal and angle-interlock woven fabrics and provided experimental validation of these models. However, the effect of shear deformation on the fabric permeability was not considered in the studies mentioned above. In recent years, researchers [19,20,21,22,23,24] have studied the permeability of two-dimensional fabrics (including single- and double-layer plain woven and non-crimp fabrics) under shear deformation through theoretical analyses, numerical simulations, and experiments, which found that shear deformation played an important role in determining the fabric permeability. Demaria et al. [25,26] present experimental results for deformed and undeformed fabrics obtained by unidirectional flow measurement, and numerical simulations for an automotive body part are carried out to illustrate the effects of fabric shearing on the filling of the composite part. Numerical methods have several advantages compared to experimental tests. Experiments are time-consuming, subjected to a high degree of variation [27,28], and can only be used to investigate existing fabrics. Weaving technology allows for the design of any type of fabric architecture, which makes a reliable numerical tool desirable.

Aside from the in-depth work on the permeability of two-dimensional fabrics and three-dimensional orthogonal and angle-interlock fabrics without shear deformation, researchers have also performed detailed analyses on the permeability of two-dimensional fabrics under shear deformation. However, the permeability of the 2.5D woven preform under shear deformation has not been studied. Compared to plain woven fabrics, the three-dimensionally interwoven fiber structure of this preform gives rise to a more complex geometry of the unit cell. The binder warp also exerts some influence on the permeability. Predicting the permeability of this structure under shear deformation is thus more challenging. Based on the results of experiment and simulation, a corrected empirical formula for predicting the permeability change of the 2.5D woven preform with shear deformation is concluded in this paper.

In this study, the 2.5D woven preform was treated as a porous media. The principle in-plane permeability and the angle between the principal permeability and the weft of the fabric were measured at four different shear angles by the radial flow method [13]. A finite element model of the unit cell was established for the four different shear angles, to take into account the changes in yarn morphology caused by the shear deformation. The effects of the volume fraction of the warp and weft yarn on the fabric permeability as well as that of shear angle were studied. The empirical equation for the preform was corrected using the simulation results. The validity of the corrected equation was confirmed experimentally. The influence of the shear angle on the fabric permeability is provided. This work could serve as reference for the RTM process and mold design of the 2.5D woven preform.

## 2. Materials and Methods

### 2.1. Experimental Materials

**Figure 1:**
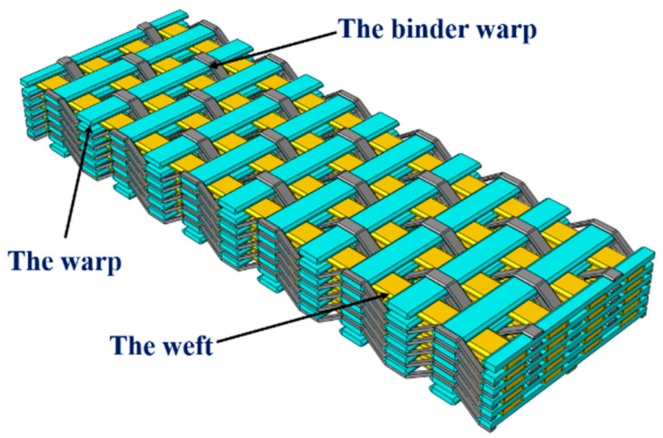
The geometry structure of a 2.5D woven preform.

The 2.5D woven preform used in this study is made by the Institute of Composite Materials, Tianjin Polytechnic University (Tianjin, China). It was composed of warp yarn (T700/12K), weft yarn (T700/12K), and binder warp yarn (T300/3K), as illustrated in Figure 1. The textile structure shown in Figure 1 is a kind of 3D fabric, which is called 3D layer to layer angle interlock fabric (also known as 2.5D fabric). This preform had a typical layer-to-layer interlocked architecture, in which the warp and weft yarns ran in the *x*- and *y*-directions, respectively, and were locked in the thickness direction by the binder warp. The warp and binder warp yarns were arranged with a ratio of 1:1 in the *x*-direction. The exact structural parameters are shown in Table 1.

**Figure 2:**
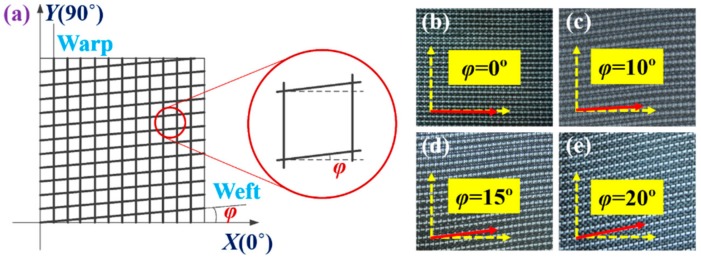
Four samples with different shear angles: (**a**) schematic of the shear Angle; (**b**) the sample with a shear angle of 0°; (**c**) the sample with a shear angle of 10°; (**d**) the sample with a shear angle of 15°; (**e**) the sample with a shear angle of 20°.

To study the effect of shear deformation on the permeability of the 2.5D woven preform, shear deformation was induced accurately on the fabrics at designated shear angles. The permeabilities of the samples under various shear angles were measured by the radial flow method. At small shear angles, the rebound in the woven preform after shear deformation rebound was not significant. However, at large shear angles, the rebound was substantial after unloading. Fabric samples with shear angles of 0°, 10°, 15°, and 20° were chosen to study the influence of the shear deformation on the permeability of the 2.5D woven preform. A circular hole is punched into the center of the sheared fabric to act as an injection point during the experiment. Sample and their specific parameters can be found in Figure 2 and Table 2 and Table 3.

### 2.2. Radial Flow Permeability Measurements

**Figure 3:**
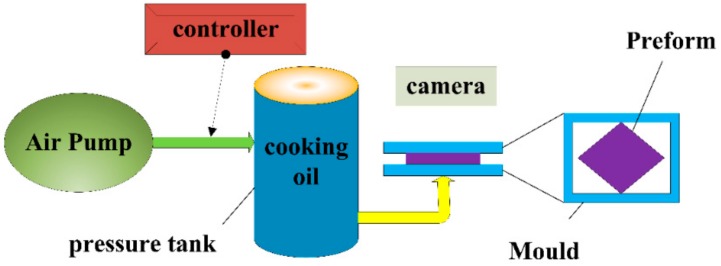
Experimental setup of the radial flow method.

To measure the permeability of the 2.5D woven preform under shear deformation, a visualizable permeability experiment was tailor-designed for this study. The upper part of the mold was a transparent acrylic glass cover. Because the yarn spacing of 2.5D fabric is relatively large, there will be negligible small extrusion between the yarns when shear deformation occurs, and the thickness variation is therefore very small. The stiffness in thickness direction is negligibly small compared with that of the acrylic glass. Thus, the tool deflection is neglected. The pre-deformed fabric was cut into a specific size and placed in the mold. The experiment was performed as shown in Figure 3. A constant pressure of 0.15 MPa was applied to the pressure tank by an air pump and a pressure controller. Under this constant pressure difference, cooking oil infiltrated the 2.5D woven preform. The entire process was recorded by a camera. To facilitate the recording and observation of the infiltration path of the cooking oil, the woven preform was marked every 10 mm in the warp and weft directions with white fiberglass threads. Glass fiber tracer yarns are designed to facilitate observation of the flow front of a liquid. As a result, glass fiber tracer yarns exist only on the surface of the fabric, the proportion of glass fiber tracer yarns is minimal (9%). Here, the effect of the tracer yarns was ignored. The coordinates of the flow front edge were read off the marks on the top panel of the mold. The flow trajectory of the liquid was recorded. Cooking oil was chosen in this case as the infiltration medium, as it was free from curing issues during the course of the experiment. As such, the permeability values obtained were more accurate. The viscosity of this cooking oil at room temperature was 1.365 *Pa.s*. The high viscosity of liquid will reduce the injection speed of liquid, which is helpful to the observation of experimental phenomena and the collection of experimental data.

**Figure 4:**
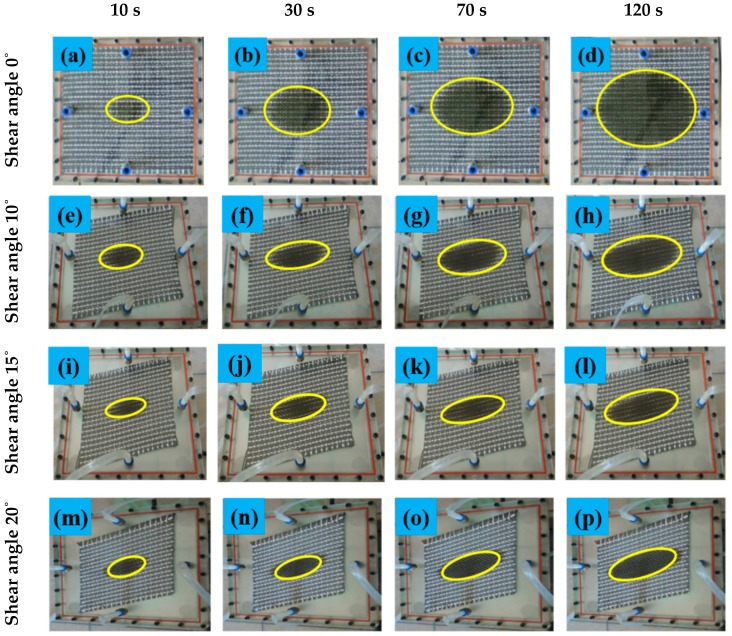
Liquid infiltration process of preform at constant pressure. (**a**,**b**,**c**,**d**) are pictures of the flow front of the liquid at different moments when the shear angle of the fabric is 0°; (**e**,**f**,**g**,**h**) are pictures of the flow front of the liquid at different moments when the shear angle of the fabric is 10°; (**i**,**j**,**k**,**l**) are pictures of the flow front of the liquid at different moments when the shear angle of the fabric is 15°; (**m**,**n**,**o**,**p**) are pictures of the flow front of the liquid at different moments when the shear angle of the fabric is 20°.

**Figure 5:**
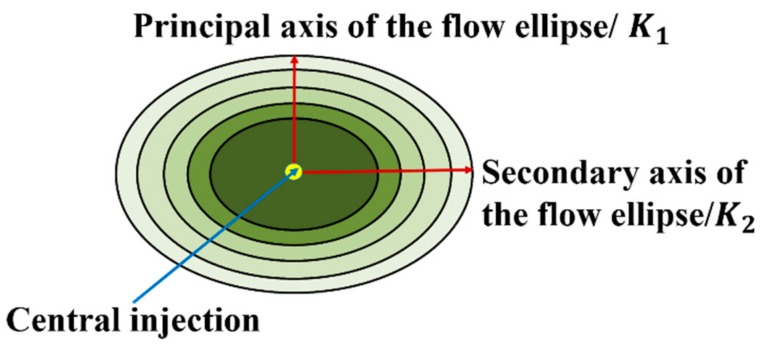
Scheme for experimental result extraction.

The radial flow method was used to measure the permeability of the fabric samples. During the experiment, the liquid gradually spread outward from the injection point to the surrounding fabrics, and an elliptical front edge of the liquid is observed. Figure 4 shows the infiltration process of the 2.5D woven preform by the cooking oil under constant pressure. The major and minor semi-axes (i.e., *a* and *b*) of the elliptical front edge of the liquid flow were extracted using the method shown in Figure 5 and were substituted into Equations (1) and (2) [1] to calculate the two principle permeabilities of the 2.5D woven preform:

(1) $$K_{1}=\frac{a}{b}\cdot \left({\left(a\cdot \sqrt{\frac{b}{a}}\right)}^{2}\cdot 2\cdot \ln\left(\frac{a\cdot \sqrt{\frac{b}{a}}}{r_{in}}-1\right)+r_{in}^{2}\right)\cdot \frac{\mu \cdot \delta }{4t\cdot \Delta p},$$

(2) $$K_{2}=\frac{b}{a}\cdot \left({\left(b\cdot \sqrt{\frac{a}{b}}\right)}^{2}\cdot 2\cdot \ln\left(\frac{b\cdot \sqrt{\frac{a}{b}}}{r_{in}}-1\right)+r_{in}^{2}\right)\cdot \frac{\mu \cdot \delta }{4t\cdot \Delta p},$$

where *a* and *b* are the lengths travelled by the fluid along the elliptical major and minor axes, and *K*_1_ and *K_2_* are the permeabilities along these directions, respectively. *r_in_* is the radius of the injection gate in the woven preform, *t* is the time of the liquid gradually spread outward from the injection point to the surrounding fabrics, and *μ*, *δ*, and Δ*p* are the fluid viscosity, fabric porosity, and the pressure difference between the injection outlet and the perimeter of the fabric, respectively.

## 3. Results and Discussion

### 3.1. Simulation Analysis

The structure of the 2.5D woven preform was rather complex. As a more intuitive and detailed account of the relationship between its configuration and permeability, the representative volume element (RVE) was used to analyze the influence of the fabric microstructure and shear deformation on the permeability by varying the structural parameters of the unit cell.

#### 3.1.1. Unit Cell Model with Shear Deformation

**Figure 6:**
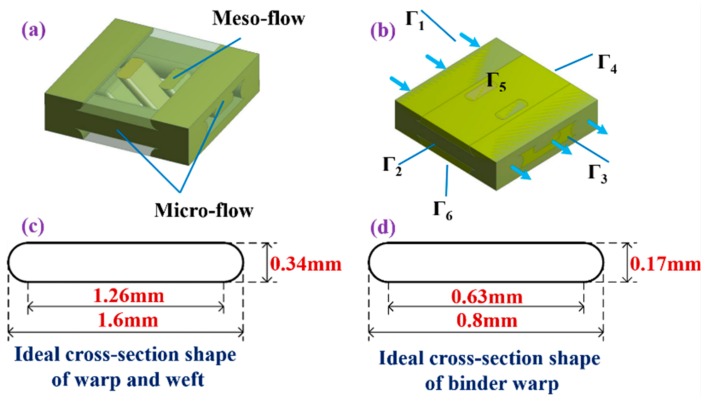
The RVE (representative volume element) of a 2.5D woven preform and its boundary conditions: (**a**) the flow of resin within RVE; (**b**) boundary conditions; (**c**) cross section parameters of warp and weft; (**d**) cross section parameters of binder warp.

Based on the finite element model of this literature [17,18,20], the 2.5D woven preform was regarded as a porous material with a definite geometry. The flow of resin in the fabric unit cell consisted of the micro-flow of resin in the interior of yarn and the meso-flow between the yarns, as depicted in Figure 6a. Shear deformation was the main type of fabric deformation, whose degree could be quantified by the shear angle. The geometric structure of the 2.5D woven preform changed after shear deformation, but the new structure still possessed a periodic pattern, making it possible to predict its permeability by the unit cell method. The cross section parameters of warp, weft, and bundling warp are shown in Figure 6c,d, respectively. The micro-flow channel in the yarn and the meso-flow channel between yarns were taken as two materials with distinct permeabilities and modelled as such using the Fluent software (Fluent 6.3.26, ANSYS, Cannonsburg, PA, USA). The pressure field was applied to the entire unit cell. The velocity field thereby obtained was substituted into Darcy’s law to find the permeability of the unit cell. The distance between the thin wall and the single cell is set as 0, and the grid of the fluid region is divided by Boolean shear. Using hexahedral mesh, the optimized mesh size is about 0.75 mm. By combining the Navier–Stokes equation and continuity equation, the pressure distribution and velocity distribution of the liquid can be obtained, and the permeability value can be obtained by substituting Darcy’s law. Based on the established 2.5D woven fabric single cell model, fluent software was used to simulate the flow between yarns and predict the single cell permeability. In this paper, a finite element model of the fabric was constructed on the unit cells with four different shear angles to investigate the variation of the permeability of the 2.5D woven preform with shear angle. Parameters of the finite element model are as listed in Table 4 and Table 5.

#### 3.1.2. Boundary Conditions and Permeability Calculation

Figure 6b shows the model of a complete unit cell. To calculate the permeability in the *x*-direction (warp-direction), the following boundary conditions were set for the unit cell: the two faces Г_1_ and Г_3_ perpendicular to the *x*-direction had constant pressures and formed a pressure gradient along this direction. The other four faces all had periodic boundary conditions. Based on Darcy’s Law, Chen et al. [14] proposed Equations (3), (4), and (5) to solve for the permeability, which could be used on plain-woven cloth. The flow velocity integral on Г3 (the outgoing plane of fluid flow) was obtained by the established finite element model. This integral was substituted into Equation (3) to solve for the *x*-direction permeability. Similarly, using the same finite element model and Equations (4) and (5), the permeabilities in *y*- and *z*-directions could be obtained. Equations (3), (4), and (5) are as follows:

(3) $$K_{x}=\frac{\mu L_{1}}{{Г}_{3}P_{0}}\iint_{{Г}_{3}}uds,$$

(4) $$K_{y}=\frac{\mu L_{2}}{{Г}_{2}P_{0}}\iint_{{Г}_{2}}vds,$$

(5) $$K_{z}=\frac{\mu h}{{Г}_{5}P_{0}}\iint_{{Г}_{5}}wds,$$

where *μ* is the fluid viscosity; *L*_1_, *L*_2_, and *h* are the lengths of the unit in the *x*-direction, *y*-direction, and *z*-direction; Г_1_ and Г_3_, Г_4_ and Г_2_, and Г_6_ and Г_5_ are the incoming and outgoing planes area of the fluid flow in the *x*-direction, *y*-direction, and *z*-direction, respectively.

### 3.2. Corrected Empirical Formula

Early on, Smith et al. [24] believed that the permeability of unidirectional fibers was only related to the fiber volume fraction and formulated a functional relation for the two quantities. However, this theoretical prediction is not applicable to other fabrics. In recent years, scientists have extensively studied the relationship between the principle permeability of various fabrics and the fiber volume fraction and shear angle, and have provided many empirical equations [1,20,21,22,23]. Unfortunately, none of these relations can accurately predict the effects of shear deformation on the permeability of the 2.5D woven preform. Based on these previous results and the structural characteristics of the 2.5D woven preform, an empirical equation predicting the effect of shear deformation on the permeability of this fabric is proposed in this paper.

The 2.5D woven preform is a special three-dimensional woven fabric with different yarn distributions in the warp and weft directions. Its two principle permeability values are not the same, even before shear deformation. Interestingly, the experimental results showed that the two principle permeabilities varied essentially in the same way as the change of shear angle. As elucidated in previous studies [1,20,21,22,23,24], the permeability of a fabric is exponentially related to its fiber volume fraction, and the shear deformation affects the permeability by changing the fiber volume fraction and the internal structure of the fabric. The latter also has a significant impact on the permeability. In view of this, a shear deformation term for the 2.5D woven preform was specifically added herein, yielding empirical Equations (6) and (7), similar to the previous ones (the trigonometric term accounts for the effects of shear deformation):

(6) $$K_{1}=\lambda_{1}\cdot \left[\xi_{1}\cdot \cos^{2}\left(\frac{90^{\circ }+\varphi }{2}\right)\right]\cdot e^{\lambda_{2}}{}^{V_{f}\left(\varphi \right)},$$

(7) $$K_{2}=\lambda_{3}\cdot \left[\xi_{2}\cdot \cos^{2}\left(\frac{90^{\circ }+\varphi }{2}\right)\right]\cdot e^{\lambda_{4}}{}^{V_{f}\left(\varphi \right)},$$

where *λ*_1_, *λ*_2_, *λ*_3_, and *λ*_4_ were four parameters to be determined, *ξ*_1_ = 0.55 and *ξ*_2_ = 0.40.

### 3.3. Prediction

**Figure 7:**
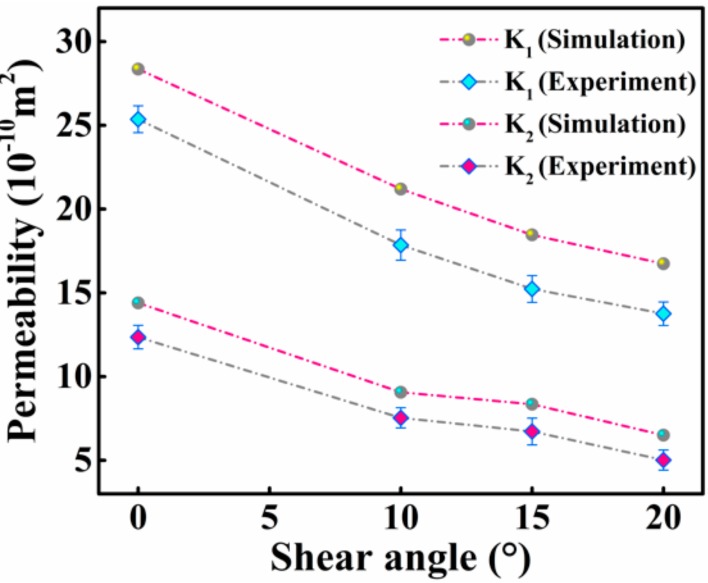
Relationship between penetration and the shear angle.

**Figure 8:**
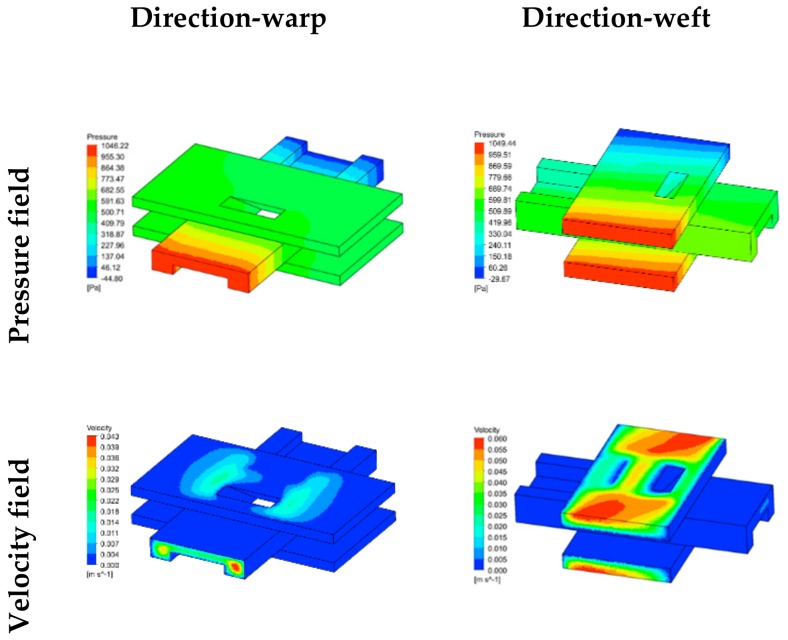
Distribution of pressure field and velocity field in flow simulation.

The relationship between the principle in-plane permeabilities and shear angle, which is revealed by the experiments and numerical simulations, is shown in Figure 7. As the shear angle increased, the principle in-plane permeabilities *K*_1_ and *K*_2_ both decreased gradually, indicating hindrance to the flow of resin in the 2.5D woven preform due to the shear deformation. This effect was augmented at larger shear deformations. The two principle in-plane permeabilities were also different in value, with the permeability in the warp-direction (*K*_2_) smaller than that in the weft-direction (*K*_1_). This shows a strong dependence of the permeability of the 2.5D woven preform on the internal structure of the fabric. The steady-state flow in a unit cell was also simulated using the Fluent program to obtain the pressure and velocity fields in the warp and weft directions, as shown in Figure 8. The analysis showed that the greatest resin flow velocity in a unit cell occurred in the channel formed by the warp, weft, and binder warp. The pressure field and velocity field changed in an unsynchronized manner in the yarn and between yarns, in that the gradient of pressure field was large in the yarn, but the flow velocity was very small. The presence of the binder warp made the warp-direction channel more complex than the weft-direction channel, and the warp-direction permeability was lower than the weft-direction. This result also proved that the permeabilities of the 2.5D woven preform were different in the warp and weft directions due to the presence of the binder warp.

The comparison of the experimental and numerical simulation results in Figure 7 shows the reliability of the latter. To determine the four unknown parameters in empirical Equations (6) and (7), the relationship between the permeability of the 2.5D woven preform and fiber volume fraction was found using numerical simulations.

#### 3.3.1. Effect of Fiber Volume Fraction on Permeability

**Figure 9:**
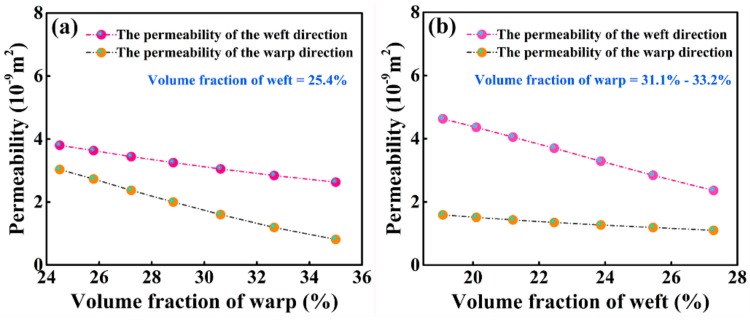
(**a**) Relationship between the volume fraction of warp and permeability; (**b**) relationship between the volume fraction of weft and permeability.

Under the condition of zero fabric deformation, the effects of the warp volume fraction (including the warp and binder warp) and weft volume fraction on the two principle in-plane permeabilities of the 2.5D woven preform were studied using a finite-element unit cell and equations based on Darcy’s law, as shown in Figure 9a,b.

As shown in Figure 9a,b, the change in the fiber volume fraction had a significant impact on the permeability of the 2.5D woven preform. As the volume fractions of the warp and weft increased, the in-plane permeabilities decreased linearly. The decline in the warp-direction permeability occurred at a higher gradient than that in the weft-direction permeability with the increase in the volume fraction of the warp, and the decline in weft-direction permeability occurred at a higher gradient than that in the waft-direction permeability with the increase in the volume fraction of the weft. At a low total fabric volume fraction, regardless of any changes in the inter-yarn spacing between the warp or weft fibers, the total fiber volume fractions of the fabric at the corresponding *x*-coordinates are identical in Figure 9a,b. A comparison revealed that there was a greater sensitivity of the warp-direction permeability to the warp volume fraction and the weft-direction permeability to the weft volume fraction.

#### 3.3.2. Effect of Shear Deformation on Magnitude of Principle Permeability

**Figure 10:**
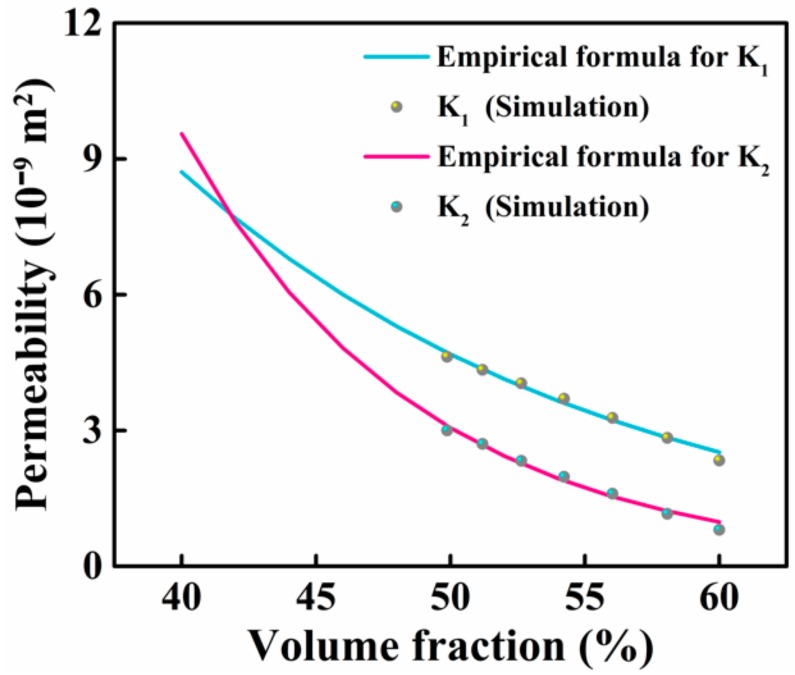
Relationship between the principle in-plane permeabilities *K*_1_ and *K*_2_ and the fiber volume fraction (*λ*_1_ = 4.16 × 10^−7^, *λ*_2_ = −6.20, *λ*_3_ = 3.64 × 10^−6^ and *λ*_4_ = −11.39 in Equations (9) and (10)).

By assuming that the thickness of the fabric did not change (as the height of the mold cavity was constant), when the 2.5D woven preform was deformed, the fiber volume fraction (*V_f_*) should increase with shear angle (*φ*) according to following relations 2.5D woven preform in Equation (8):

(8) $$V_{f}(\varphi )=\frac{V_{f}{}_{,init}}{\cos(\varphi )},$$

where the initial fiber volume fraction *V_f,init_* of the 2.5D woven preform was 50.8%. In the case that the shear angle of the 2.5D woven preform does not change and only its fiber volume fraction changes, functional Equations (9) and (10), which relate the permeability solely to the fiber volume fraction, were obtained by removing the shear effect terms in Equations (6) and (7). Using the functional relation between the permeability of the 2.5D woven preform and the fiber volume fraction in Figure 7 and Figure 8, the four parameters (*λ*_1_, *λ*_2_, *λ*_3_ and *λ*_4_) in Equations (9) and (10) were determined by data fitting with MATLAB. As shown in the fitting results of Figure 10, the two principle permeabilities decreased exponentially as the fiber volume fraction increased, and their values were clearly different. Equations (9) and (10) are as follows:

(9) $$K_{1}=\lambda_{1}\cdot e^{\lambda_{2}}{}^{V_{f}},$$

(10) $$K_{2}=\lambda_{3}\cdot e^{\lambda_{4}}{}^{V_{f}}.$$

**Figure 11:**
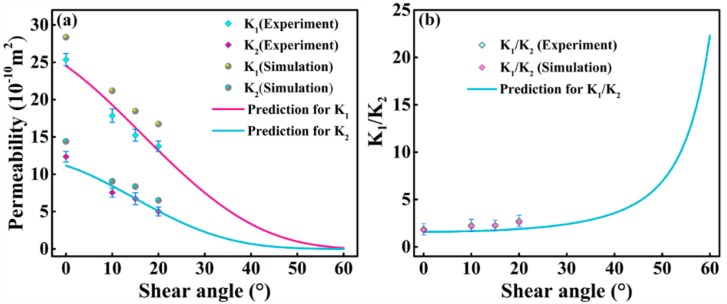
(**a**) Relationship between the principle in-plane permeabilities *K*_1_ and *K*_2_ and the shear angle (*λ*_1_ = 4.16 × 10^−7^, *λ*_2_ = −6.20, *λ*_3_ = 3.64 × 10^−6^ and *λ*_4_ = −11.39 in Equations (7) and (8)); (**b**) relationship between the ratio of the principle permeabilities and the shear angle.

When the 2.5D woven preform underwent shear deformation, the two principle in-plane permeabilities changed accordingly. Using Equations (6) and (7) and *λ*_1_, *λ*_2_, *λ*_3_ and *λ*_4_ obtained by fitting, the relation between the principle in-plane permeabilities *K*_1_ and *K*_2_ and the shear angle was obtained, as shown in Figure 11a. Apparently, the permeability of the 2.5D woven preform was sensitive to the shear deformation, as it decreased rapidly with the increase in the shear angle. Compared to the effects of the fiber volume fraction on the permeability, the shear deformation fundamentally damaged the internal geometry of the preform, thereby altering the channels of fluid flow between yarns, even to the extent that the flow channel became discontinuous. At shear angles below 10°, the decrease in *K*_1_ was rapid, while that in *K*_2_ was gentler. At shear angle above 10°, the drops in *K*_1_ and *K*_2_ were both rapid. At shear angles above 40°, both principle permeabilities approached zero very quickly, as the flow passages in the woven preform were almost completely destroyed (i.e., the fiber volume fraction of the 2.5D woven preform was almost 1).

To further study the effect of shear deformation on the permeability of the 2.5D woven preform, Equation (11) was derived by taking the ratio of Equations (6) and (7), which provides the functional dependence of the ratio of the principle in-plane permeabilities on the shear angle, as plotted in Figure 11b. The experimental results, numerical simulation results, and the curve predicted with the empirical formula presented in this paper clearly show the same trend: as the shear angle increased, the value of *K*_1_/*K*_2_ increased slowly. Comparing Figure 11a,b, the absolute difference (*K*_1_ − *K*_2_) between the two principle permeabilities of the 2.5D woven preform decreased with the increase in shear angle, while their ratio (*K*_1_/*K*_2_) increased slowly. The gradual increase in the ratio between the principle permeabilities (*K*_1_/*K*_2_) indicated a more uneven in-plane permeability at larger shear angles. If the mold is not adjusted accordingly, the fabric will not be thoroughly infiltrated. Equation (11) is as follows:

(11) $$\frac{K_{1}}{K_{2}}=\eta_{1}e^{\eta_{2}V_{f}\left(\varphi \right)},\mathrm{Where}\;\eta_{1}=\;\frac{\lambda_{1}}{\lambda_{3}},\eta_{2}\;=\;\lambda_{2}-\lambda_{4}.$$

#### 3.3.3. Effect of Shear Deformation on Direction of Principle Permeability

The angle between the warp and weft yarn is altered in a shear-deformed 2.5D woven preform, which has a considerable impact on the permeability of the fabric. In addition to the changes in the magnitudes of the principle permeabilities, their directions are also deflected.

**Figure 12:**
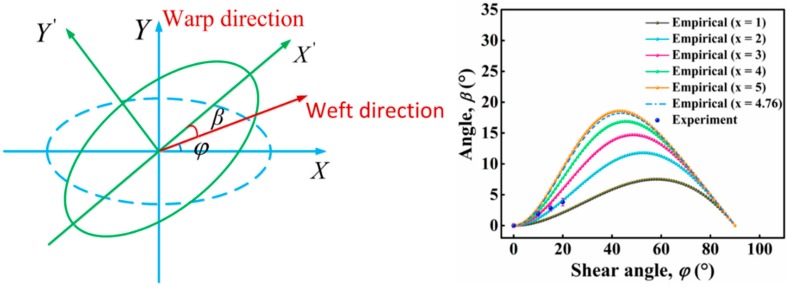
(**a**) Theoretical model for permeability testing of the fabric after shear deformation; (**b**) relationship between difference (*β*) between the shear angle (*φ*).

Assuming that the warp direction of the 2.5D woven preform remains unchanged after shear deformation, the angle between the weft before and after deformation is *φ* (shear angle). The principle permeability shifts from the *X*~*Y* frame to the *X’*~*Y’* frame, and the angle between *X’* and the shear-deformed weft is *β* (the difference between the shear angle and the angle of deflection in the direction of the principle permeability), as shown in Figure 12a. From the tests on the 2.5D woven preform with four different shear angles (as shown in Figure 4), the angle of deflection in the direction of the principle permeability was found to increase with the shear angle. This means that the shear deformation also changed the direction of the principle in-plane permeability of the fabric. The principle permeabilities no longer followed the *X*~*Y* direction but were deflected by a certain angle. This experimental result confirmed the theoretical assumption made at the beginning of this paragraph. Figure 12b gives the variation in the angle of deflection for the major semi-axis of the ellipse at the four shear angles of 0, 10, 15, and 20°. The deflection in the major semi-axis of the ellipse increased with shear angle, i.e., there was greater deflection in the direction of principle permeability.

To better analyze the influence of shear deformation on the direction of the principle permeability of a fabric, Endruweit [21] proposed an empirical formula (Equation (12)) relating *β* (the difference between the shear angle and the angle of deflection angle in the direction of principle permeability) and *φ* (shear angle) of two-dimensional fabrics:

(12) $$\begin{array}{l} \beta =\frac{90^{\circ }-\varphi }{2}\left(1-\sin^{x}\left(90^{\circ }-\varphi \right)\right),\mathrm{Where}\;x=5{\left({\left(\frac{K_{1}}{K_{2}}\right)}_{\varphi =0}-1\right)}^{-1}. \end{array}$$

The variation of *β* with *φ* was plotted using this empirical equation, as shown in Figure 12b. As shear angle increased, the angle of deflection in the direction of the principle permeability also increased gradually and was always larger than the shear angle. Based on the definition of *x* in Equation (12) and the two principle permeabilities measured at a 0° shear angle, *x* was found to be 4.76, which yielded the prediction curve in Figure 12b. Evidently, the value of *β* measured in the experiments deviated quite significantly from the predicted curve plotted using the empirical formula. As also noted previously [21], this empirical formula is not based on rigorous theoretical derivations. Since the 2.5D woven preform used in this paper was quite different in the fabric structure from those used previously [21,23], the value of *x* in the empirical formula must be re-determined. In Figure 12b, at *x* = 2, the experimentally found *β* was in better agreement with the value predicted by the empirical formula. This means the empirical formula could accurately predict the relationship between *β* and the shear angle *φ* for the 2.5D woven preform, but the value of *x* must be determined specifically for the fabric based on its structure.

## 4. Conclusions

In this study, a finite element model of the unit cell was established for the four different shear angles, to take into account the changes in yarn morphology caused by the shear deformation. The effects of the volume fraction of the warp and weft yarn on the fabric permeability as well as that of shear angle were studied. The empirical equation for the preform was corrected using the simulation results. The validity of the corrected equation was confirmed experimentally. The influence of the shear angle on the fabric permeability is provided. 

The experiments have shown that the shear deformation of 2.5D woven preform induces a change of permeability, and the permeability principal values and the orientation of the permeability principal axes of 2.5D woven preform as a function of the shear angle have been carried out. Corrected empirical formulas for the accurate prediction of the permeability of 2.5D woven preform under shear deformation, in which undetermined parameters are determined by the permeability data of undeformed 2.5D woven preform, were presented and their accuracy has been confirmed by experimental data. The results of corrected empirical formulas were as follows: (1) the shear deformation affected both the magnitude and direction of the principle permeability in the woven preform. Larger shear angles gave rise to smaller principle permeabilities and larger deflections in the direction of the principle permeability. (2) The difference (*K*_1_ − *K*_2_) between the two principle permeabilities of the 2.5D preform decreased as the shear angle increased, while their ratio (*K*_1_/*K*_2_) increased. At shear angles above 40°, both principle permeabilities approached zero rapidly.

## Figures and Tables

**Table 1:** Structural parameters of 2.5D woven preform.

Parameter	Value
the fineness of the stitching warp yarn	T300(3K)
the fineness of weft	T700(12K)
The layer number of fabric	6 layers
the density of stitching warp	3.0 piece/cm/layer
the density of the lining warp	3.0 piece/cm/layer
the density of the warp	3.0 piece/cm/layer

**Table 2:** Sizes of shear pre-deformed samples.

Sample Number	Shear Angle (°)	Sample Size (mm)
S-1	0	250 × 250
S-2	10	210 × 210
S-3	15	198 × 198
S-4	20	186 × 186

**Table 3:** The fiber volume fraction of the 2.5D woven preform.

Shear Angle (°)	The Fiber Volume Fraction
0	50.8%
10	51.8%
15	52.4%
20	54.0%

**Table 4:** Structural parameters of 2.5D woven preform RVE (representative volume element).

Parameter	Value (mm)
Warp (weft) width	1.6
Warp (weft) height	0.34
Binder warp width	0.8
Binder warp height	0.17
Lateral distance between warp (weft) yarns	3
Vertical distance between warp (weft) yarns	0.68
Lateral distance between binder warp yarns	3
Vertical distance between binder warp yarns	0.68

**Table 5:** Structural parameters of 2.5D woven preform RVE.

Shear angle (°)	Lateral Distance between Warp (Weft, Binder Warp) Yarns (mm)
0	3
10	2.94
15	2.91
20	2.82
